# Randomised controlled trial of local corticosteroid injections for carpal tunnel syndrome in general practice

**DOI:** 10.1186/1471-2296-11-54

**Published:** 2010-07-29

**Authors:** Cyriac Peters-Veluthamaningal, Jan C Winters, Klaas H Groenier, Betty Meyboom-de Jong

**Affiliations:** 1 Department of General Practice, University Medical Center Groningen, Groningen, the Netherlands

## Abstract

**Background:**

Carpal tunnel syndrome is caused by entrapment of the median nerve and results in pain, tingling and numbness in the wrist and hand. It is a common condition in general practice. Effectiveness of treatment by intracarpal corticosteroid injection has never been investigated in general practice. The objective of this study was to determine if corticosteroid injections for carpal tunnel syndrome provided by general practitioners are effective.

**Methods:**

In this study 69 participants with a clinical diagnosis of carpal tunnel syndrome were recruited from 20 general practices. Short-term outcomes were assessed in a randomised, placebo-controlled trial. Long-term results were assessed in a prospective cohort-study of steroid responders.

Participants were randomised to intracarpal injections of 1 ml triamcinolonacetonide 10 mg/ml (TCA) or 1 ml NaCl (placebo). Non-responders to NaCl were treated with additional TCA injections. Main outcomes were immediate treatment success, mean score of the Symptom Severity Scale (SSS) and Functional Status Scale (FSS) of the Boston carpal tunnel questionnaire, subjective improvement and proportion of participants with recurrences during follow-up. Duration of follow-up was twelve months.

**Results:**

The TCA-group (36 participants) had better outcomes than the NaCl-group (33 participants) during short-term assessment for outcome measures treatment response, mean improvement of SSS-score (the mean difference in change score was 0.637 {95% CI: 0.320, 0.960; p < 0.001}) and FSS-score (the mean difference in change score was 0.588 {95% CI: 0.232, 0.944; p = 0.002}) and perceived improvement (p = 0.01). The number to treat to achieve satisfactory partial treatment response or complete resolution of symptoms and signs was 3 (95% CI:1.83, 9.72).

49% of TCA-responders (17/35) had recurrences during follow-up. In the group of TCA-responders without recurrences (51%, 18/35) outcomes for SSS-score and FSS-score deteriorated during the follow-up period of 12 months (resp. p = 0.008 and p = 0.012).

**Conclusions:**

Corticosteroid injections for CTS provided by general practitioners are effective regarding short-term outcomes when compared to placebo injections. The short-term beneficial treatment effects of steroid injections deteriorated during the follow-up period of twelve months and half of the cohort of steroid-responders had recurrences.

**Trial registration:**

Current Controlled Trials ISRCTN53171398

## Background

Carpal tunnel syndrome (CTS) is caused by entrapment of the median nerve at the wrist and symptoms consist of paresthesias and numbness in the area of median nerve innervation. Frequently pain in the hand and wrist is present, sometimes radiating to more proximal areas of the arm. Most cases are idiopathic, sometimes there are underlying factors causing compression of the median nerve (e.g. oedema during pregnancy)[1-3]. The role of occupational and recreational hand use in causation remains controversial. The exact role of overuse in the aetiology of CTS remains unclear, although there is some evidence that regular and prolonged use of hand-held vibratory tools and prolonged and highly repetitive flexion and extension of the wrist increases the risk[1-3]. In the Netherlands (7.000.000 working people) it has been estimated that every year 370.000 days of absence from work result from disability caused by CTS. This corresponds with 26,5 million euro of costs per year caused by absence from work due to CTS [2].

CTS is a frequently encountered condition with an annual incidence rate of 1.8 per 1000 (males 0.9/1000, females 2.8/1000) in general practice in the Netherlands and the prevalence rate in the general population is 5.8% (9% for women and 0.6% for men)[4-6]. The average list size of general practitioners in the Netherlands is 2350 patients.

There is no golden diagnostic standard for CTS and in practice guidelines it is advised to establish the diagnosis using a combination of symptoms, signs and electrophysiological testing[2,7].

CTS can be treated with oral analgesics, splinting, injections with corticosteroids or surgery. A Cochrane review investigating local corticosteroid injection for carpal tunnel syndrome showed that steroid injection provides greater improvement in symptoms one month after injection than placebo injection, but significant symptom relief of steroid injection beyond one month could not be demonstrated[8]. The risk of adverse events for steroid injection therapy for CTS has been estimated to be less than 0,1%[9]. In another Cochrane review addressing efficacy of other non-surgical treatments oral steroids, splinting, ultrasound, yoga and carpal bone mobilisation showed to be of short-term benefit[10]. A third Cochrane review comparing surgical to non-surgical treatment concluded that surgical treatment of carpal tunnel syndrome relieves symptoms significantly better than splinting[11].

In general practice in the Netherlands 25% of patients with a clinical diagnosis of CTS are referred to neurologists for further evaluation and treatment[5]. It is not known what percentage of patients with CTS is treated conservatively and which operatively.

If corticosteroid-injection provided by general practitioner proves to be effective and safe, it could have important advantages for individual patients (less waiting-time and the availability of this treatment modality in the proximity of the patient) and healthcare-system (treatment in primary care would be more cost-effective).

We therefore decided to conduct a randomised, double blind, placebo controlled trial to investigate efficacy and safety of corticosteroid injections provided by their general practitioner for patients with a clinical diagnosis of CTS.

## Methods

This trial is part of a larger study called the Groningen Hand and Wrist Injection Therapy Trial (HAWITT) in which efficacy and feasibility of steroid injections for carpal tunnel syndrome, de Quervain's tenosynovitis and trigger finger in primary care was evaluated. In this report the results for carpal tunnel syndrome are described.

The trial was approved by the Medical Ethics Committee of University Medical Centre Groningen (METc 2002/020c).

### Setting

Patients were recruited from the practices of 20 general practitioners in the northern part of the Netherlands.

### Patient recruitment and in- and exclusion criteria

Patients presenting to the participating general practitioners with symptoms and signs suggestive of carpal tunnel syndrome were eligible for inclusion. Exclusion criteria were thenar atrophy, being less than years of age, presence of contraindications for corticosteroid injection (hypersensitivity to corticosteroids, local skin infection), prior treatment for CTS in the last six months with steroid injection or surgery, traumatic or neoplastic origin of symptoms, inability to fill in follow-up forms or absence of self-determination in the participant. In participants with bilateral symptoms general practitioners were instructed to include the hand with the most severe complaints. After applying in- and exclusion criteria, written informed consent was obtained from participants by their general practitioner.

### Interventions and injection technique

Participants received one or two intracarpal injections with either 1 ml triamcinolonacetonide 10 mg/ml (experimental intervention) or 1 ml NaCl 0.9% (control intervention). One millilitre of either TCA or NaCl was injected just to the ulnar side of the palmaris longus tendon, proximal to the wrist crease. The needle was aimed toward the carpal tunnel at a 10- to 20-degree angle of entry. If there were no paresthesias during insertion of the needle, the trial solution was injected[1]. All general practitioners involved in the study were offered a two-hour course on the technique of injection therapy, using an arm phantom for instruction.

### Randomisation and allocation concealment

For the randomisation procedure an electronic online randomization tool developed by G. Urbaniak (http://www.randomizer.org, accessed on 22.12.2002) was used. Block randomisation was realised by creating 7 sets of blocks of 10 random numbers. Even numbers corresponded with active trial medication and uneven numbers with placebo to ensure equal numbers of allocation to active and placebo treatment. Treatment allocation was written on a paper and enclosed in an opaque and sealed envelope. After inclusion of a patient a pharmacy assistant at a remote location (who was not involved in the study) was contacted, who then drew an envelope and sent the allocated trial medication to the injecting general practitioner.

### Study design, blinding and bail out treatment

Every patient with typical signs and symptoms of carpal tunnel syndrome presenting to one of the participating general practitioners was asked to participate in the trial. As an aid in establishing the diagnosis of carpal tunnel syndrome a list of clinical criteria for CTS of the American Academy of Neurologists and a modified version of a hand diagram developed by Katz et al. were provided[7,12]. After applying inclusion and exclusion criteria, assessment of baseline clinical characteristics took place by the patient's own general practitioner, who also performed the blinded assessment of the short-term follow-up two weeks after the intervention. In order to guarantee blinding of short-term outcome assessment (after randomisation) the trial medication was injected one week after inclusion by another independent general practitioner. If the result of the first injection was not satisfactory in the participant's opinion, the participants were given a second injection by the other independent general practitioner one week later. One week after the last injection with the trial medication the participants were instructed to return to their own general practitioner for assessment of short-term outcomes. Because a placebo look-alike of the triamcinolonacetonide injection suspension could not be manufactured, blinding was realised by applying the injection while the participant was blindfolded.

### Bailout treatment

If during short term outcome assessment the response to the blinded injection(s) was insufficient according to agreement between the patient and general practitioner, blinding was discontinued and the trial centre was asked whether injected trial medication consisted the active substance (TCA) or control treatment (NaCl). Participants who were randomized to TCA with no response to blinded injections were referred to secondary care for operative treatment and not included in the long-term analysis.

In case of insufficient response after injection of NaCl, one or two additional injections with TCA (bail-out treatment) with weekly intervals were given without blinding. In case of insufficient response to one or two bailout injections, participants were referred to secondary care for operative treatment and not included in the long-term follow-up analysis. Introducing bailout treatment for non-responders to NaCl was required, as the medical ethics committee considered it to be unethical to leave patients, who received placebo treatment with no improvement in symptoms after intervention, untreated.

### Outcomes measurements

Baseline assessment consisted of recording of demographic and disease-specific characteristics of participants to identify differences in prognostic indicators between the two intervention groups.

During short-term assessment the following primary outcome measurements were recorded:

1. Direct treatment response (based on consensus between physician and patient):

• 0 = no response

• 1 = partial response, but not satisfactory, warranting further treatment

• 2 = partial response, satisfactory, not warranting further treatment

• 3 = complete resolution of symptoms and signs

2. Improvement as perceived by patient:

• -2 = much worse

• -1 = worse

• 0 = not better/not worse

• + 1 = better

• + 2 = much better

3. Symptom severity was assessed by using the Symptom Severity Scale (SSS) and functional disability by using the Functional Status Scale (FSS), which are both part of the Boston Carpal Tunnel Questionnaire (BCTQ). The BCTQ is a patient-reported outcome measure for CTS and has been tested for validity, reliability and responsiveness. Psychometric properties of the BCTQ have been described extensively elsewhere[13]. The SSS has 11 questions, the FSS 8 questions and both use a five-point scale. Each scale generates a final score (sum of individual item scores divided by number of items), which ranges from 1 to 5. Higher SSS and FSS scores correlate with more severe symptoms and functional impairment respectively.

4. proportion of participants with recurrences requiring repeat TCA-injections or referral to secondary care for operative treatment during the follow-up period of 12 months.

5. The secondary outcomes of side effects and adverse events were systematically recorded (qualitatively and quantitatively) at short-term assessment and during follow-up.

Follow up measurements were performed by sending questionnaires to participants 1, 3, 6 and 12 months after the last injection and consisted of the same outcome measures as during short term assessment except for direct treatment response.

Data regarding the number of recurrences (requiring repeat steroid injection or referral to secondary care for operative treatment) and handling of recurrences during the follow-up phase were extracted from the electronic health records of participants.

### Sample size and data analysis

Calculations of sample size were based on a two-sided alpha of 0.05, a statistical power of 0.90. The proportion of participants treated with steroid injection with satisfactory response or complete resolution of symptoms after two injections was expected to be at least 60%, extrapolated from prior prospective studies[14,15]. Adequate treatment response to placebo treatment was expected to be 20%. Based on these calculations we aimed to recruit 34 patients for each treatment group. Analysis was planned according the intention to treat principle. For continuous data the student T-test was used if the distribution was normal and Mann-Witney U test if there was not a normal distribution. For categorical data Fisher's exact test was used. Friedmann's test was used to compare repeated observations on the same subjects and to test if the distributions are the same across repeated measures if a non-normal distribution of outcome data was suspected. Significance was accepted at a probability value of < 0.05.

To calculate the Number Needed to Treat the formula NNT = 1/ARR was used, where: ARR (Absolute Risk Reduction) = CER (Control Group Event Rate) - EER (Experimental Group Event Rate). The Event Rate was the proportion of participants with a partial satisfactory response, not warranting further treatment or complete resolution of symptoms and signs for the outcome direct treatment response.

Missing follow-up values were imputed based on the available follow-up scores using the EM algorithm, assuming that missing data occurred completely at random (MCAR) [16]. Data were analysed using the statistical software SPSS version 14 (SPSS Inc Chicago, Illinois, USA).

## Results

During a period of 33 months (February 2003 to October 2005, follow-up finished in October 2006) 69 participants who fulfilled the inclusion were recruited by 20 general practitioners in 20 general practices. At baseline assessment the two groups were found to be comparable regarding potentially prognostic indicators and differed only in mean duration of symptoms. The median duration of symptoms was 13 weeks in the NaCl-group (P_25 _= 7, P_75 _= 50) and 26 weeks in the TCA-group (P_25 _= 8, P_75 _= 52) (see table 1). In 66 of the 69 (96%) of the included patients the hand diagram was rated as classical or probable CTS.

**Table 1:** baseline characteristics of study population

		NaCl (n = 33)	TCA(n = 36)
**mean age (SD)**		57.60 (40.30)	56,.5 (15.14)

**sex (female/male)**		26/7	27/9

**median duration of symptoms (weeks) (P25, P75)**		13 (7,50)	26 (8.52)

**repetitive movements of hands**		10/22	15/18

**affected hand/arm (right/left)**		21/9	18/14

**dexterity (right/left)**		32/0	31/3

**quality of symptoms:**	a. dull aching discomfort arm/hand	25	28

	b. weakness/clumsiness hand	22	23

	c. paraesthesias hand	30	35

	d. nocturnal complaints	28	32

	e. presence of relieving factors	25	25

	f. presence of provocative factors	30	31

			

**score Katz hand diagram**			

	classic	12	11

	probable	19	22

	unlikely	1	2

			

**mean BCTQ symptom score (SD)**		2.82 (0.79)	2.89 (0.78)

**mean BCTQ functional score (SD)**		2.35 (1.05)	2.48 (1.02)

**comorbidity**			

	diabetes	0	1

	hypothyroidism	2	2

	rheumatoid arthritis	1	1

	pregnancy	0	1

NaCl = NaCl 0.9% (saline)

TCA = triamcinolonacetonide 1 mg/ml

BCTQ = Boston Carpal Tunnel Questionnaire

After randomisation 36 patients were allocated to TCA and 33 to NaCl (one participant who was originally randomised to NaCl, was mistakenly allocated to TCA).

### Short-term efficacy

**Figure 1:**
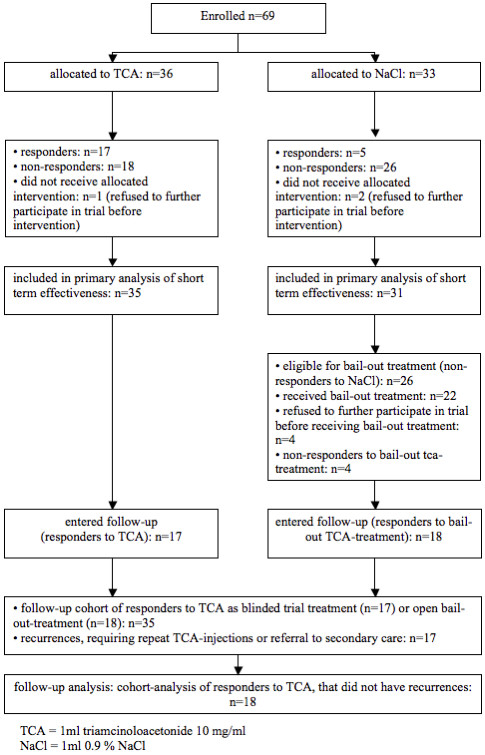
flow of patients during intervention phase

The results of primary outcomes one week after the last injection as compared to baseline measurement are displayed in table 2. Three participants refused further participation in the study after randomisation for unknown reasons. Therefore they did not receive the allocated intervention and were not analysed (figure 1).

**Table 2:** short-term results after one or two injections of NaCl or TCA

		NaCl	TCA	p-value
**direct treatment response**	no response	17	9	

	partial response, not satisfactory	9	9	

	partial response, satisfactory	5	11	

	complete resolution of symptoms	0	6	

				0.013

**mean score BCTQ**		2.815 (0.795)	2.872(0.785)	
**symptom severity scale**				
**before intervention (SD)**				

**mean score BCTQ**		2.529(0.847)	1.948(0.779)	
**symptom severity scale**				
**after intervention (SD)**				

**mean score BCTQ**		2.353(1.045)	2.456(1.024)	
**functional status scale**				
**before intervention (SD)**				

**mean score BCTQ**		2.366(1.0988)	1.881(0.810)	
**functional status scale**				
**after intervention (SD)**				

**change in mean score**		0.286(0.554)	0.924(0.710)	< 0.001
**BCTQ symptom severity scale (SD)**				

**change in mean score**		-0.135(0.557)	0.575(0.838)	p = 0.002
**BCTQ functional status scale (SD)**				

**patient perceived improvement**	much worse	1	0	

	worse	2	1	

	not better not worse	17	9	

	better	10	9	

	much better	2	15	

				0.01

NaCl = NaCl 0.9% (saline)

TCA = triamcinolonacetonide 1 mg/ml

BCTQ = Boston Carpal Tunnel Questionnaire

11 participants received one blinded TCA injection and 24 received two blinded TCA injections. Of the participants that received one injection 10 responded to treatment (91%) and of the 24 that received two injections 7 responded (30%).

The TCA-group showed better direct treatment response (p = 0.013), perceived improvement (p = 0.01) and more improvement than the NaCl-group in the outcomes SSS BCTQ score (from 2.872 to 1.948 in the TCA group versus from 2.815 to 2.529 in the NaCl group) and FSS BCTQ score (from 2.456 to 1.881 in the TCA group versus from 2.353 to 2.366 in the NaCl group). The mean difference in change score was 0.637 (95% CI: 0.320, 0.960; p < 0.001) for the SSS BCTQ and the mean difference in change score was 0.588 (95% CI: 0.232, 0.944; p = 0.02) for the FSS BCTQ. The Number Needed to Treat to achieve satisfactory partial treatment response or complete resolution of symptoms and signs was 3 (95% CI: 1.83, 9.72).

### Long-term efficacy

All non-responders to blinded intervention were required to be treated with (non-blinded) TCA-injections and all non-responders to TCA (blinded and as bail-out treatment) were referred to secondary care for operative treatment. Therefore, it was decided to present the long-term follow-up data of the effects of corticosteroid injections as a report of the cohort of patients that had responded to treatment with TCA.

51% of the 69 included patients (35/69) entered the follow-up period. 51% of these TCA-responders (18/35) did not report any recurrences during follow-up and 49% of TCA-responders (17/35) had recurrences.

**Figure 2:**
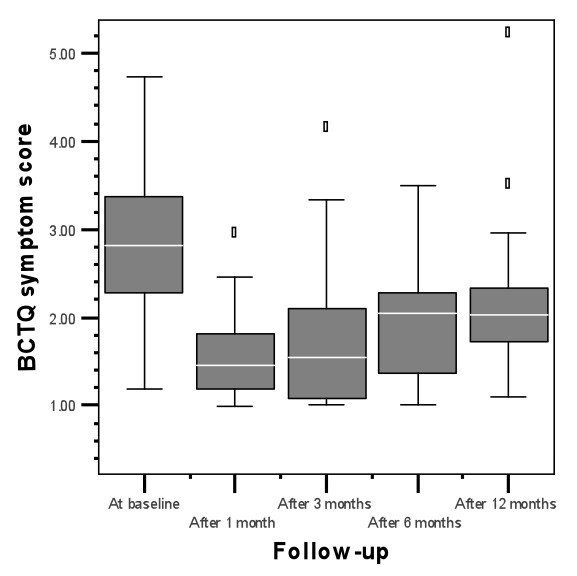
BCTQ symptom score of responders to TCA during follow-up

**Figure 3:**
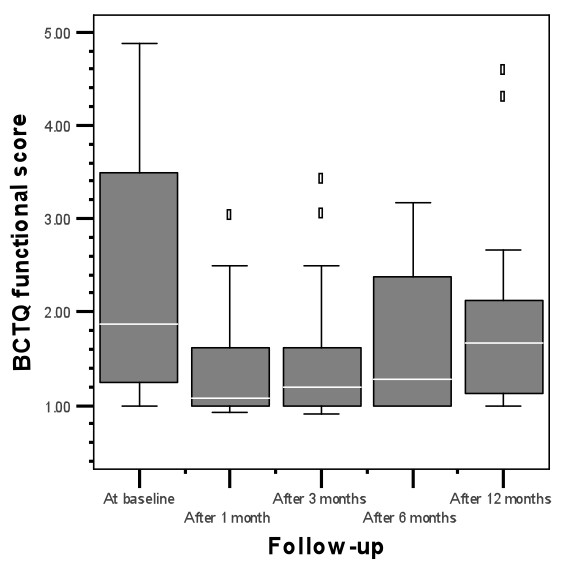
BCTQ functional score of responders to TCA during follow-up

In the cohort that remained free of recurrences the short term beneficial treatment effects of steroid injection(s) deteriorated during follow-up: main outcomes BCTQ SSS (1.45, 1.55, 2.05 and 2.03 at resp. 1, 3, 6 and 12 months follow-up; p = 0.008) and BCTQ FSS (1.08, 1.19, 1.28 and 1.66 at resp. 1, 3, 6 and 12 months follow-up; p = 0.012) increased during the entire follow-up period of twelve months (figure 2 and 3, table 3), however they did not reach the pre-intervention levels (the median score for the BCTQ SSS was 2.90 and for the BCTQ FSS 2.50 at baseline for the participants treated with TCA-injections).

**Table 3:** long-term results of responders to tca that did not have recurrences during follow-up

	Follow-up				
	
	1 month	3 months	6 months	12 months	**p-value**^**1**^
**Median score BCTQ symptom severity scale(min, max)**	1.45	1.55	2.05	2.03	
	(0.99, 2.90)	(1.00, 4.10)	(1.00, 3.49)	(1.09, 5.18)	0.008
	N = 18	N = 18	N = 18	N = 18	

**Median score BCTQ functional status scale (min, max)**	1.08	1.19	1.28	1.66	
	(0.93, 2.99)	(0.91, 3.38)	(1.00, 3.16)	(1.00, 4.53)	0.012
	N = 18	N = 18	N = 18	N = 18	

1. Friedman test

BCTQ = Boston Carpal Tunnel Questionnaire

In the TCA-responders that had recurrences 27 recurrences occurred in 17 participants. 9 participants had 1 recurrence, 6 participants 2 recurrences and 2 participants had 3 recurrences. 15 of the 27 recurrences (11 participants) were treated with steroid injection (7 participants with one injection, 4 participants with 2 injections). None of the participants with recurrences were treated with splinting. 12 (12 participants) of the 27 recurrences (17 participants) were referred to secondary care for operative treatment.

### Complications of treatment

There were no serious adverse events reported during short-term and long-term assessment. The most frequent reported side effects that had occurred within one week after blinded interventions and bailout treatment were steroid-flare (a delayed post injection transient increase in pain which has been attributed to crystal-induced synovitis): 14 events, hot flushes: 7 events, vasovagal symptoms: 3 events and menstrual irregularities: 2 events.

## Discussion

### Summary of main findings

This is the first randomised controlled trial assessing efficacy of steroid injections for carpal tunnel syndrome in general practice.

Our results indicate that steroid injections applied by trained general practitioners are effective regarding short-term outcomes when compared to placebo injections. The effect size at short-term (one week after last injection) assessment was substantial with a number needed to treat of three to achieve satisfactory partial treatment response or complete resolution of symptoms and signs. The difference with placebo injections in mean change scores were resp. 0.637 for the SSS BCTQ and 0.588 for the FSS BCTQ. The mean scores of the symptom and functional subscale of the Boston Carpal Tunnel Questionnaire after steroid injection changed positively with respectively 0.92 and 0.58. Both values are higher than the threshold of 0.8 (SSS BCTQ) and 0.5 (FSS BCTQ) for clinical importance using patient satisfaction as a criterion as determined by Leite et al [13]. Although the TCA-group had a much longer duration of symptoms at baseline assessment, the short-term outcomes were better.

Long-term effectiveness is less clear, since long-term data were only available for the cohort of participants who responded to TCA during the study and blinding was discontinued if there was no response to the intervention at short-term assessment.

Scores for BCTQ-SS and BCTQ-FSS deteriorated during the follow-up period of 12 months, although they did not reach pre-intervention levels.

Furthermore 17 (49%) of responders to TCA had recurrences during the follow-up period of 12 months and in this group 11 participants required treatment with additional steroid injections and 12 ultimately had to be referred to secondary care for operative treatment.

### Comparison with existing literature

If we compare our results to findings of two high quality randomised controlled studies performed in secondary care by Dammers et al. and Armstrong et al. it appears that response rate in our study is less (50% compared to 70% in study by Dammers and 70% in the study by Armstrong), but duration of treatment response, recurrence rates and timing of recurrences were similar[14,15]. The smaller response rate could partly be explained by the type and dosage of steroid that was used: Dammers et al used 40 mg of methylprednisolon (which is a higher dosage of a steroid with the same potency as triamcinolonacetonide, which was used by us) and Armstrong used 6 mg of bethametason (a more potent steroid than triamcinolonacetonide). A second explanation could be the fact that we used rigorous allocation concealment and randomisation procedures, since bias due to inadequate allocation concealment and randomisation can lead to overestimation of treatment effects. Thirdly it could have been possible that in less clear-cut cases the diagnosis of carpal tunnel syndrome was not certain and therefore steroid injection were less effective (although the scores of the Katz-hand diagram suggest that general practitioners can diagnose carpal tunnel syndrome reliably on clinical grounds). A final possible explanation for the smaller response rate to steroid injections in our study could be the long duration of symptoms at baseline for the steroid-group in our study (76 weeks) as compared to the study by Dammers (32 weeks) and Armstrong (39% of the steroid group had symptoms for less than one year).

### Strengths and the limitations of this study

Strong points in our study were that randomisation, allocation concealment and blinding procedures were rigorous (with blinding of patient and outcome assessors) and that with the Boston Carpal Tunnel Questionnaire we used a valid and reliable patient-based outcome measurement tool.

Since we excluded patients with thenar wasting, it might have been that our study consisted of milder cases than previous studies, which only studied secondary care patient populations.

Our trial protocol did not include any nerve conduction studies, because the aim of our study was to investigate effectiveness of steroid injection for participants with a clinical diagnosis of CTS as established by a general practitioner. Nevertheless, the clinical characteristics and results of hand diagram scores of participants (table 1) show that a large proportion (96%) of our study population had typical features of CTS and that therefore general practitioners seem to identify classical cases of CTS reliably. Although practice guidelines for CTS suggest that Nerve Conduction Studies (NCS) are important to establish the diagnosis of CTS, NCS are a controversial issue since there is no gold diagnostic standard for CTS and NCS have shown to have certain limitations (mainly lack of sensitivity and standardized protocols) and correlations between NCS and clinical outcome measures are weak to moderate, a phenomenon also known as the "clinical-neurophysiologic paradox" [2,7,17].

Due to the decision of the medical ethics committee we had to discontinue blinding in our study if there was no response to trial intervention at short-term assessment, since it was considered unethical to leave patients with symptoms of carpal tunnel syndrome untreated during the follow-up period of one year. One could argue that introduction of bailout treatment for placebo non-responders has led to less robust long-term data and this therefore on the other hand would justify from an ethical perspective a randomised controlled trial (with a follow-up period of one year) without the use escape-treatment for non-responders to placebo-treatment. Two other randomised controlled trials that investigated efficacy of steroid injections for CTS in secondary care were faced with the same dilemma [14,15].

### Implications for future research or clinical practice

In our opinion steroid injection into the carpal tunnel is a safe, easy to learn and to apply and a relatively inexpensive therapeutic intervention. Also response to steroid injection could be helpful in establishing the diagnosis of carpal tunnel syndrome. Our study indicates that general practitioners can reliably identify patients with carpal tunnel syndrome using symptoms, signs and a self-administered hand diagram.

Although our study has certain limitations, the design and setting of our study leads to conclusions that are generizable for the population of patients presenting to their general practitioner with a clinical diagnosis of carpal tunnel syndrome. Therefore we feel that initial treatment by general practitioners with steroid injections in cases of CTS with a typical history and without thenar wasting is justified. If there is no response after two injections or if recurrences occur, nerve conduction studies may be warranted before surgical therapy is considered.

Although we observed only a few minor side effects and no adverse events occurred in our study, studies addressing safety of corticosteroid injections for CTS provided by general practitioners using larger sample sizes are needed.

## Conclusions

The results of our study suggest that in patients presenting to their general practitioner with a clinical diagnosis of CTS intra-carpal injection with one or two injections with 1 ml triamcinolonacetonide 10 mg/ml is effective with respect to short-term outcomes when compared to placebo-injections.

Long-term effectiveness is less clear: the achieved treatment effects seem to diminish slowly in half of the cohort of patients that responded to steroid injections during the 12 months after the intervention and recurrences occurred in the other half of the cohort of steroid responders.

## Competing interests

The authors declare that they have no competing interests.

## Authors' contributions

CPV is the guarantor and was responsible for daily project-management, trial-design, trial-logistics, data-collection and text of the paper and was supervised during these processes by JCW and BMJ. JCW initiated the study, obtained funding, contributed to the trial design and text of the paper. KHG performed the statistical analyses and BMJ contributed to the design of the trial and text of the paper. All authors read and approved the final manuscript.

## Pre-publication history

The pre-publication history for this paper can be accessed here:

http://www.biomedcentral.com/1471-2296/11/54/prepub
